# Efficacy of Laropiprant in Minimizing Brain Injury Following Experimental Intracerebral Hemorrhage

**DOI:** 10.1038/s41598-017-09994-5

**Published:** 2017-08-25

**Authors:** Abdullah Shafique Ahmad, Monique Mendes, Damian Hernandez, Sylvain Doré

**Affiliations:** 1 Department of Anesthesiology, University of Florida, Gainesville, FL USA; 2 Center for Translational Research in Neurodegenerative Disease and McKnight Brain Institute, University of Florida, Gainesville, FL USA; 3 Departments of Neurology, Psychiatry, Pharmaceutics, Psychology, and Neuroscience, University of Florida, Gainesville, FL USA

## Abstract

Intracerebral hemorrhage (ICH) is one of the most devastating and disabling forms of stroke, yet effective treatments are still lacking. Prostaglandins and their receptors have been implicated in playing vital roles in ICH outcomes. Recently, laropiprant, a DP1 receptor antagonist, has been used in combination with niacin to abolish the prostaglandin D_2_-(PGD_2_)-induced flushing. Here, we test the hypothesis that laropiprant limits bleeding and rescues the brain from ICH. Wildtype (WT) and DP1^−/−^ mice were subjected ICH and neurologic deficits and hemorrhagic lesion outcomes were evaluated at 72 hours after the ICH. To test the therapeutic potential of laropiprant, WT mice subjected to ICH were treated with laropiprant at 1 hour after the ICH. The putative effect of laropiprant on limiting hematoma expansion was tested by an *in vivo* tail bleeding cessation method and an *ex vivo* coagulation method. Finally, the roles of laropiprant on gliosis and iron accumulation were also investigated. A significant decrease in the injury volume was observed in DP1^−/−^ as well as laropiprant-treated WT mice. The tail bleeding time was significantly lower in laropiprant group as compared with the vehicle group. Significantly lower Iba-1 and Perls’ iron staining in DP1^−/−^ and laropiprant-treated WT groups were observed. Altogether, the data suggest that laropiprant treatment post-ICH attenuates brain damage by targeting primary as well as secondary injuries.

## Introduction

Intracerebral hemorrhage (ICH) is one of the most fatal stroke subtype and account for about 10–30% of all stroke patients^1, 2^. Only about 20% of people who survive ICH achieve functional independence at 6 months post-ICH^3–5^. The fatality rate at 1 month post-ICH is about 40% and the incidence is increasing every year due to the increasing number of aging population^1, 5^. ICH can lead to primary or secondary brain injury which is irrespective of the cause of the injury^6–10^. Primary injury is caused by the hemorrhage and hematoma expansion resulting in the increased intracranial pressure and mechanical injury to the tissue^11^. Secondary brain injury is the result of the physiological and pathological response to the hematoma^7^. Thus, therapeutic strategies have been developed to target primary and secondary brain injury following ICH^6, 11–14^. Therapies against primary injury target limiting the intracranial bleeding, hematoma expansion, and intracranial pressure^11^; secondary injuries are countered by targeting excitotoxicity, oxidative stress, and neuroinflammation^7, 11, 15, 16^.

An important feature of the pathophysiological response to ICH is the inflammatory cascade triggered by the degeneration and rupture of the blood vessels and hematoma formation. This inflammatory cascade involves the prostaglandin (PG), a family of lipid mediators, as well as other pathways^16–18^. Prostaglandins execute their effects through their respective receptors. The role of the prostaglandins and their specific receptors in acute neurologic conditions is actively being investigated. Interestingly, data so far suggests a paradoxical role of these prostaglandins and their receptors based on the nature, type, and location of the injury^18–25^.

PGD_2_ is the most abundant prostaglandin in the brain and exerts most of its biological effects by binding to the DP1 receptor. This receptor couples with Gs protein and activates adenylyl cyclase, resulting in an increase in cAMP which is reported to be involved with increases blood flow and platelet aggregation inhibition^26^. We have previously shown that DP1 receptor activation augments cerebral blood flow, which formed the basis for our hypothesis that absence of the DP1 receptor or its blockade by laropiprant, a selective DP1 receptor antagonist, would minimize brain injury and neurologic deficits following intracerebral hemorrhage. Interestingly, laropiprant, also known as MK-0524, has been used in pre-clinical and clinical settings alone or in combination with niacin^27, 28^ essentially to attenuate the PGD_2_-induced vasodilation that results in hot flashes on skin (a common undesirable side effect of niacin treatment). In humans, laropiprant has been found to be well tolerated, and has very decent bioavailability and a long half-life^26, 29^.

In the current study, a single dose of collagenase was injected into the striatum of WT and DP1^−/−^ mice to induce ICH. Neurologic deficits and lesion volumes were assessed at 72 hours. To test whether the beneficial effects of DP1 receptor inhibition could be the result of a reduction in hematoma expansion, non-ICH WT and DP1^−/−^ mice and laropiprant-treated WT mice were tested for tail bleeding time and *ex vivo* coagulation. Finally, the role of laropiprant treatment and DP1 receptor deletion was also assessed in gliosis through the analysis of Iba-1 and GFAP immunoreactivity, and iron content through Perls’ staining.

## Materials and Methods

### Mice

All procedures were performed in accordance with the guidelines of the National Institutes of Health (Bethesda, MD) and were approved by the Institutional Animal Care and Use Committee at the University of Florida. Adult male WT and DP1^−/−^ C57BL/6 mice of 11–13 weeks old (24–28 g) were bred and maintained in our animal facility. The DP1^−/−^ mice grow normally and have no gross abnormalities in behavior and brain macroscopic vasculature anatomy indices when compared to WT mice^30, 31^. Mice were given food and water *ad libitum* and housed under controlled reversed light cycle conditions (23 ± 2 °C; 12-hour light/dark cycle).

### Randomization, Exclusion, and Blinding

Randomization was performed by a person who was not involved in the study and who had no knowledge of experimental groups. The preset exclusion criteria included any mouse with apparent abnormal conditions (e.g. dermatitis, red eye or discharge, abdominal or whisker abnormalities, signs of infection, etc.), ear puncture during placement in the stereotactic setup, bleeding upon drilling or on needle insertion, or an animal reaching a moribund condition requiring euthanasia. The number of mice used for the WT, Vehicle, DP1^−/−^, and laropiprant groups were 12, 10, 12, and 10 respectively. The number of animals used for tail bleeding assay were 10, 8, 10, 10, and 5 for the WT, Vehicle, DP1^−/−^, WT with laropiprant treatment, and DP1^−/−^ with laropiprant treatment groups respectively. For the *ex vivo* coagulation study 5 mice/group were used. For *in vivo* thrombosis, five mice per group were used. The surgeon and investigators performing neurobehavioral testing were blinded to the experimental groups. Additionally, all histologic outcomes were quantified in a blinded manner.

### Collagenase-induced ICH Model and Laropiprant Treatment

Mice were anesthetized with isoflurane (3% for induction, 1 to 1.5% for maintenance) and their heads were immobilized on a stereotaxic frame. A small incision was made at the overlying skin over the skull and a single unilateral, intrastriatal injection of collagenase VII-S (0.04 units in 0.2 μL saline, Sigma) was given at the following stereotactic coordinates relative to bregma: 0.5 mm anterior, 2.4 mm lateral, 3.2 mm from dura^19^. Rectal temperature was monitored and maintained at 37.0 °C ± 0.5 °C throughout the surgery. After the surgery, mice were moved to a temperature and humidity regulated recovery chamber for 2 hour then moved to their home cages and were allowed to survive up to 72 hours after ICH.

In a separate cohort of similarly ICH-induced WT mice, a single dose of 0.4 mg/kg laropiprant was given at 1 hour after the collagenase injection. Mice were allowed to survive up to 72 hours after ICH. The dose chosen is based on reports showing the effect of this compound in attenuating PGD_2_ or niacin-induced cutaneous vasodilation in mice^29^. Laropiprant is reported to have a half-life up to approximately 18 hours as well as good bioavailability^26^.

### Neurological Deficit Scores (NDS)

The neurologic deficits were assessed at 72 hours post-ICH using the 24-point scale^19, 32^. The tests included body symmetry, gait, climbing, circling behavior, front limb symmetry, and compulsory circling. Each test was graded from 0 to 4, establishing a maximum deficit score of 24.

### Histology and Immunohistochemistry

At 72 hours after ICH, mice were deeply anesthetized and transcardially perfused with phosphate-buffered saline (PBS, pH 7.4) followed by fixation with 4% paraformaldehyde. The brains were quickly harvested and processed for histology and immunohistochemistry using Leica CM 1850 cryostat. The mounted sections were stained with Cresyl violet to estimate the lesion volume following the detail protocol described by us recently^19, 33, 34^. To identify microgliosis and astrogliosis sections were processed for immunohistochemistry as described by us previously^33^ by using the following primary antibodies: ionized calcium-binding adaptor protein 1 (Iba1; 1:1,000; Wako, Richmond, VA) and glial fibrillary acidic protein (GFAP; 1:1,000; Dako, Carpinteria, CA) respectively. For the ferric iron content, Perls’ iron staining was performed by incubating the sections in a mixture containing equal ratio of 2% hydrochloric acid and 2% potassium ferrocyanide for 20 min, followed by counterstaining with nuclear fast red as previously reported by us^34^. All stained sections were imaged by using ScanScope CS and analyzed with ImageScope software (Aperio Technologies, Inc., Vista, CA).

### Tail Bleeding Monitoring

To determine whether DP1 inhibition plays a role on platelet adhesion at the site of injury, a tail bleeding test was performed following the protocol we described earlier^35, 36^. This techniques is widely used to determine the ability of platelets to form a hemostatic plug^37, 38^. Briefly, the mice were either non-treated or given an i.p. injection of the vehicle (1% DMSO) or 0.4-mg/kg laropiprant. At 30 minutes from the injection, the mice were anesthetized and 2 mm of the tail tip was excised. The tail was immediately placed in warm PBS (37.0 ± 0.5 °C) and the time until bleeding ceased was recorded.

### *Ex Vivo* Thrombus Formation

A modified *ex vivo* clot formation model, as we^35^ as well as Prasad *et al*.^39^ have described in the past, was used to test whether laropiprant can prevent hematoma expansion. Whole blood from non-ICH WT mice was harvested by cardiac puncture and 200 µL of the blood was mixed and vortexed with 600 µL of the vehicle or 600 µL of 30.6 µM laropiprant. The mixture was kept at room temperature for 2 minutes, followed by centrifugation for 30 seconds at 4000 *g*. The tubes were gently removed and the amount of uncoagulated supernatant was quantified.

### Statistics

Statistical analyses were performed using Prism 5 (GraphPad, San Diego, CA). Neurologic deficit scores were analyzed by the non-parametric Kruskal–Wallis test followed by Dunn’s multiple comparison test and are presented as medians with interquartile ranges (25^th^ and 75^th^ percentiles). The remaining data sets were checked for differences in variances between groups and normality, and a one-way ANOVA test was performed, followed by Newman-Keuls multiple comparison test or an unpaired two-tailed Student’s t test with Welch’s correction. Data are expressed as Mean ± SD with *P* < 0.05 considered statistically significant.

### Data availability

The datasets generated during and/or analyzed during the current study are available from the corresponding author on reasonable request.

## Results

### Absence or Blockade of PGD_2_ DP1 Receptor Limits Hemorrhagic Brain Damage

**Figure 1:**
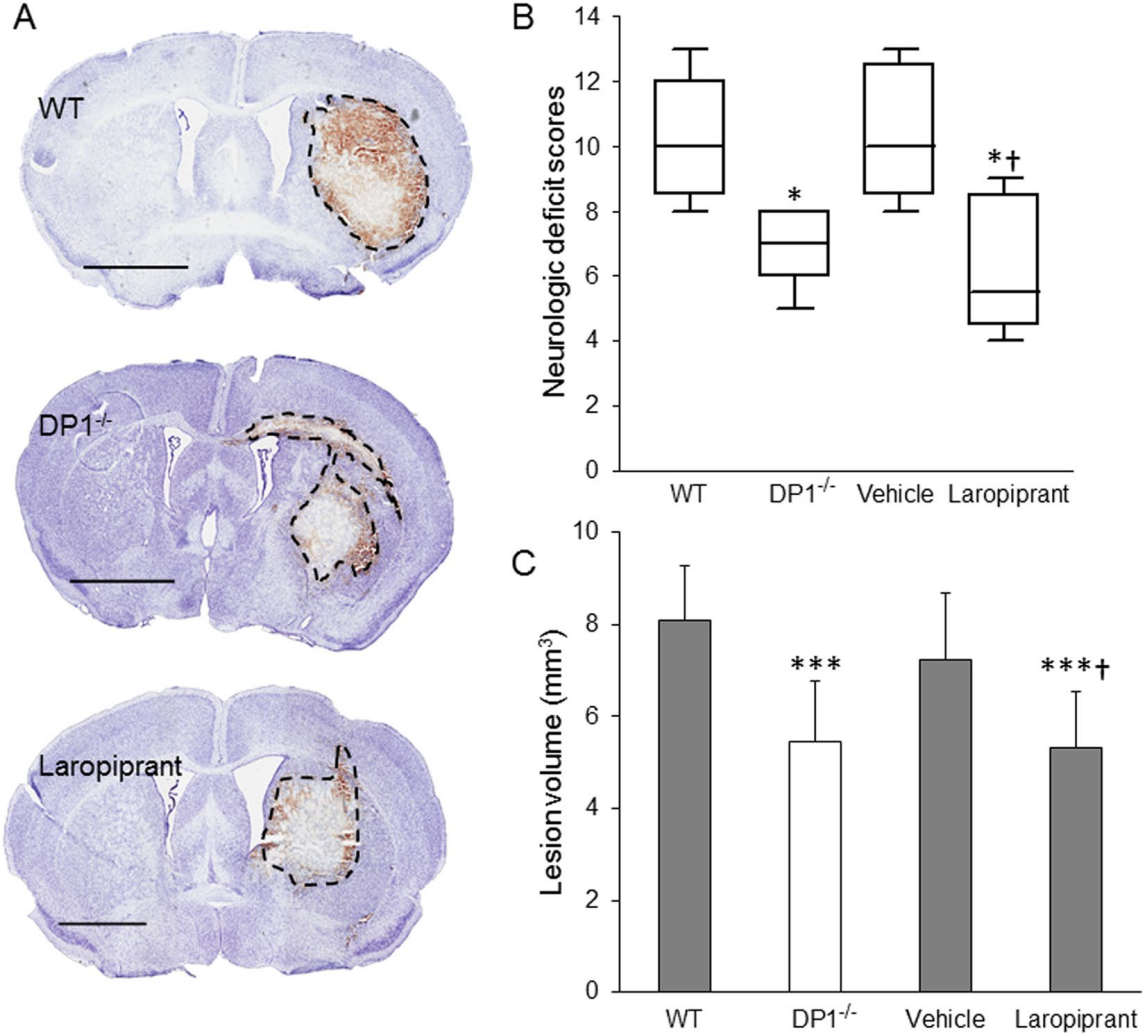
Laropiprant treatment or deletion of DP1 receptor attenuates ICH outcomes. WT and DP1^−/−^ mice were subjected to ICH by giving a single intrastriatal injection of collagenase. Another cohort of similarly ICH-induced WT mice were given vehicle or laropiprant at 1 hour after the ICH. Neurological deficits were scored at 72 hour post-ICH; thereafter, brains were harvested by perfusion and fixation. Cryosections were stained with Cresyl violet for lesion volume quantification. (**A**) Representative macrographs of CV-stained sections obtained from WT (n = 9), DP1^−/−^ (n = 9), and laropiprant (n = 8) groups. Black dotted line encompasses lesion area. (**B**) ICH-induced neurological deficits were significantly lower in DP1^−/−^ and laropiprant-treated WT groups. (**C**) DP1^−/−^ mice and the WT mice treated with laropiprant also resulted in a significant decrease in the lesion volume as compared with the WT or vehicle (n = 8) treatment groups. *P < 0.05, ***P < 0.001 compared with the WT group; ^†^ *P* < 0.05 compared with the vehicle group. Scale bar = 2 mm.

As expected, the collagenase injection in WT and DP1^−/−^ mice resulted in significant brain damage. The lesion volume calculated in DP1^−/−^ was 32.74 ± 16.31% lower than the lesion volume in WT mice. Similarly, the NDS were also significantly lower in DP1^−/−^ as compared to the WT mice (Fig. 1).

Then, to determine if pharmacological inhibition of the receptor will lead to similar outcomes, ICH-induced mice were given a single dose of 0.4 mg/kg laropiprant 1 hour after ICH. Interestingly, laropiprant was able to attenuate the NDS by 40.32 ± 18.78% and lesion volume by 26.59 ± 16.80% as compared with the vehicle treated group (Fig. 1).

### Laropiprant Treatment or Deletion of DP1 Receptor Attenuates Bleeding

**Figure 2:**
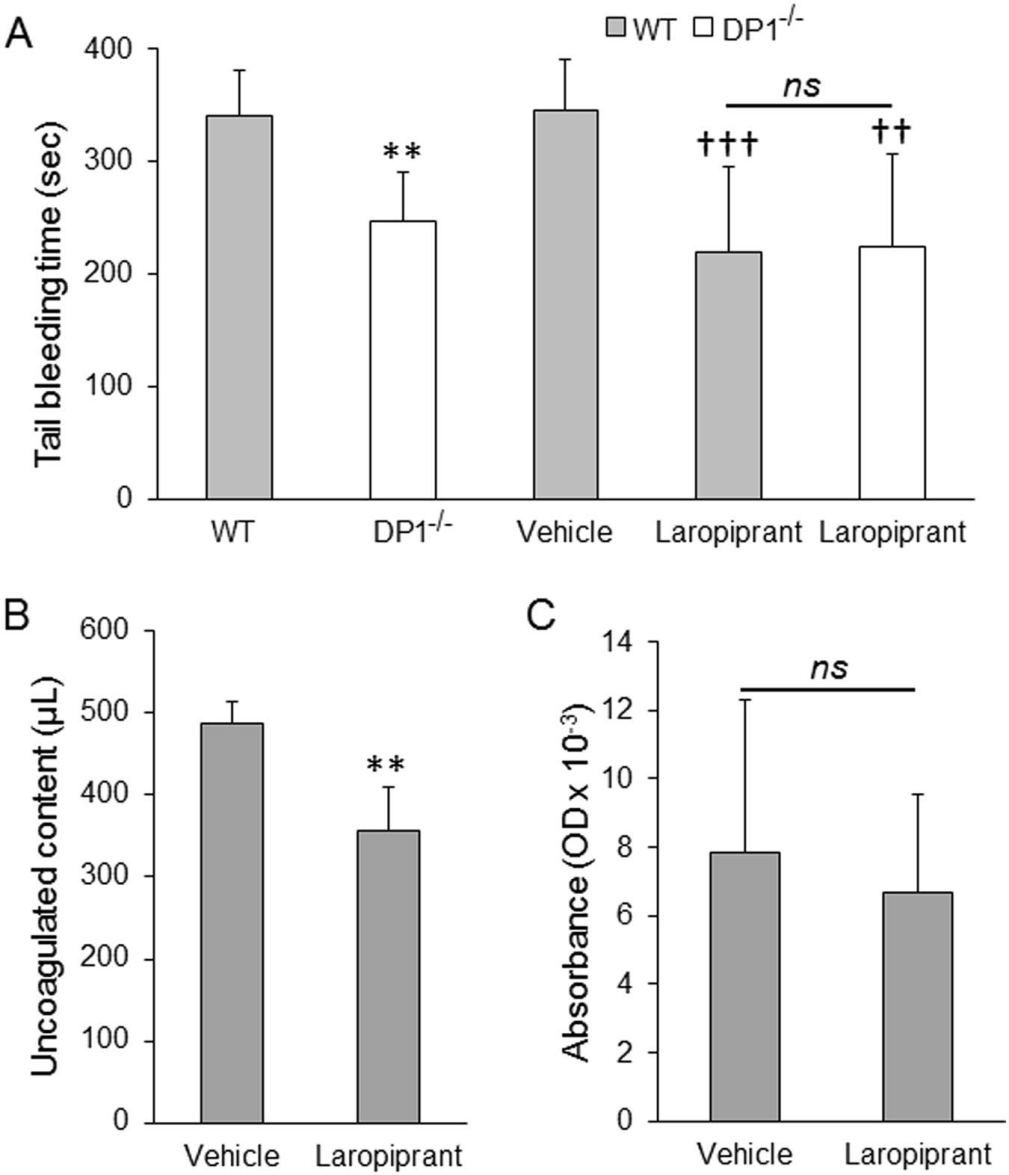
Laropiprant treatment affects hemostasis under hemorrhagic condition. (**A**) To determine blood coagulation *in vivo*, the tail tip of anesthetized mice was excised, and bleeding time was recorded. To determine the effect of laropiprant on bleeding, vehicle or laropiprant was given intraperitoneal and a tail bleeding test was performed 30 min after the injection. DP1^−/−^ and laropiprant-treated WT groups exhibited a significantly faster cessation of bleeding (n = 8–10/group). Interestingly, the DP1^−/−^ mice treated with laropiprant (n = 5) exhibited no difference as compared with either the DP1^−/−^ mice or the laropiprant treated WT mice. (**B**) To determine blood coagulation *in vivo*, blood was harvested by cardiac puncture from WT mice and mixed with vehicle or laropiprant. After incubation and centrifugation, the uncoagulated content was found to be significantly lower in the laropiprant-treated group (n = 5/group). (**C**) Chronic use of laropiprant *in vivo* did not induce microhemorrhage or microthrombus formation, suggesting that its effect on coagulation is only under hemorrhagic condition (n = 4/group, *P* = 0.68). **P < 0.01 compared with the WT group; ^††^ *P* < 0.01, ^†††^ *P* < 0.001 when compared with the vehicle group; ns = non-significant.

To determine whether the beneficial effects of laropiprant are due to its role in limiting the bleeding, we monitored tail bleeding time. We found that bleeding caused by tail clipping ceased significantly faster in groups that were treated with laropiprant or lacked the DP1 receptor. The bleeding time was 36.45 ± 21.79% and 26.51 ± 13.31% lower in laropiprant treatment and DP1^−/−^ groups, respectively (Fig. 2A). To further evaluate whether the effects of laropiprant are mediated through the DP1 receptor, the tail bleeding assay was also performed in the DP1^−/−^ mice treated with laropiprant. Interestingly, there was no significant difference between the bleeding time in DP1^−/−^ and DP1^−/−^ treated with laropiprant (247.4 ± 43.2 minute vs 224.6 ± 82.7 minute; *P* = 0.48). Then, considering that laropiprant use could putatively lead to microhemorrhage or microthrombosis *in vivo*, non-ICH mice were given intraperitoneal injection of 0.4 mg/kg/day laropiprant for 7 days, and brains were then harvested and their hemoglobin content was quantified. Interestingly, there was no significant difference (*P* = 0.68) in the hemoglobin content in vehicle or laropiprant-treated groups (Fig. 2D).

### Laropiprant Treatment Facilitates *Ex Vivo* Coagulation

To determine whether the beneficial effects of laropiprant are due to its influence on coagulation, which would thus limit hematoma expansion, whole blood was incubated with laropiprant. Treatment of the whole blood with laropiprant led to larger clot formation *ex vivo*. The uncoagulated content after the clot formation was quantified and found to be 26.77 ± 11.02% lower in the laropiprant-treated group (Fig. 2B).

### DP1 Receptor Deletion Augments Microgliosis

**Figure 3:**
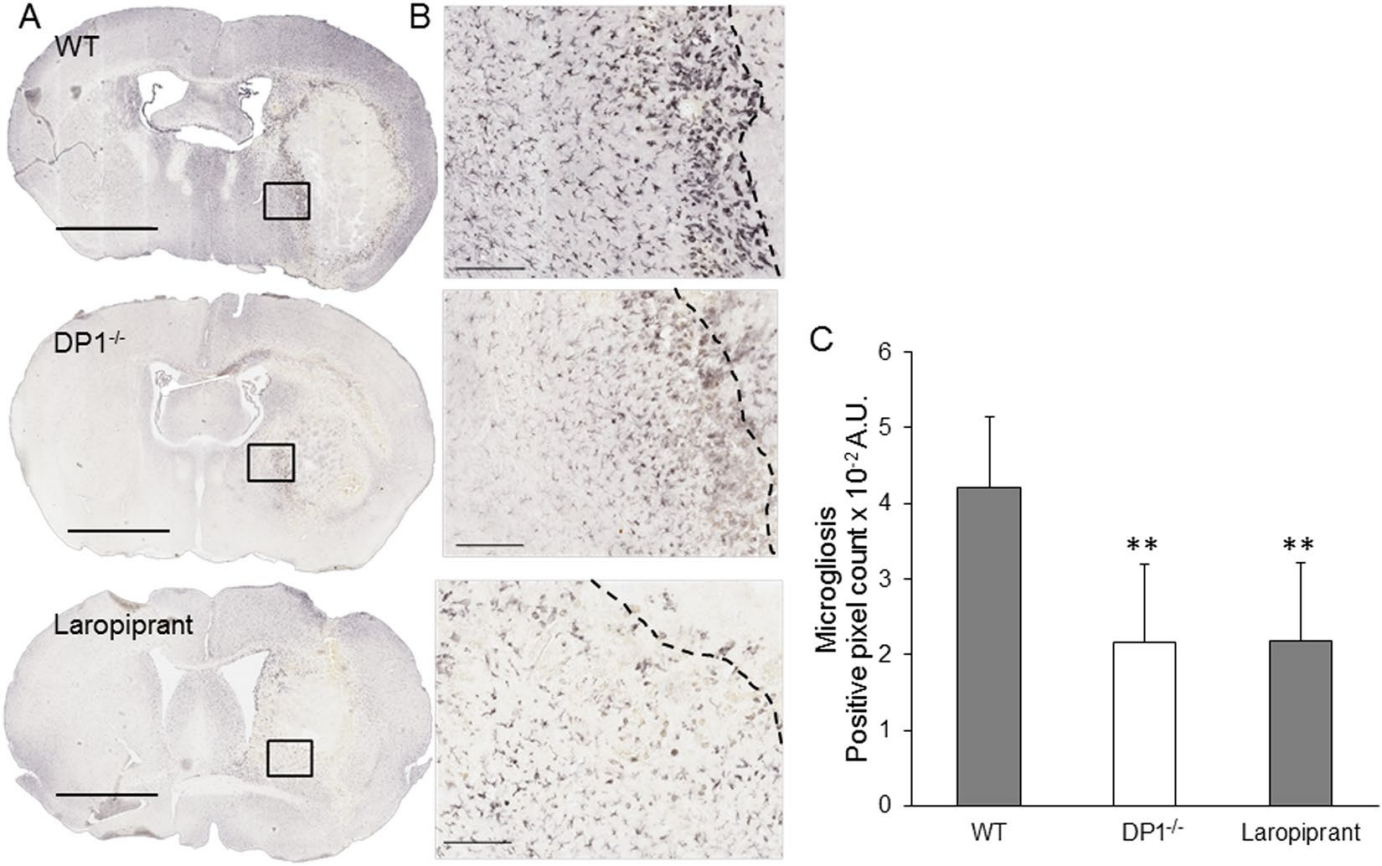
Laropiprant attenuates microgliosis. The brain sections were immunostained for Iba-1 immunoreactivity to monitor microgliosis. (**A**) Representative macrographs at 1x showing Iba-1 immunoreactivity in WT, DP1^−/−^, and WT mice treated with laropiprant. Scale bar = 2 mm. (**B**) The regions in black boxes in panel A were magnified at 10x. The black dotted line demarcates the lesion core from the perihematoma area. Scale bar = 200 µm. The perihematoma and the intact area in WT mice exhibit two distinct morphologies of microglia. The microglia closer to the perihematoma exhibited amoeboid morphology whereas, those in the intact area show activated morphology. (**C**) Quantification of the Iba-1 immunoreactivity shows significant reduction in Iba-1 immunoreactivity in DP1^−/−^ and WT treated with laropiprant. **P < 0.01 compared with the WT group. n = 6–7/group.

**Figure 4:**
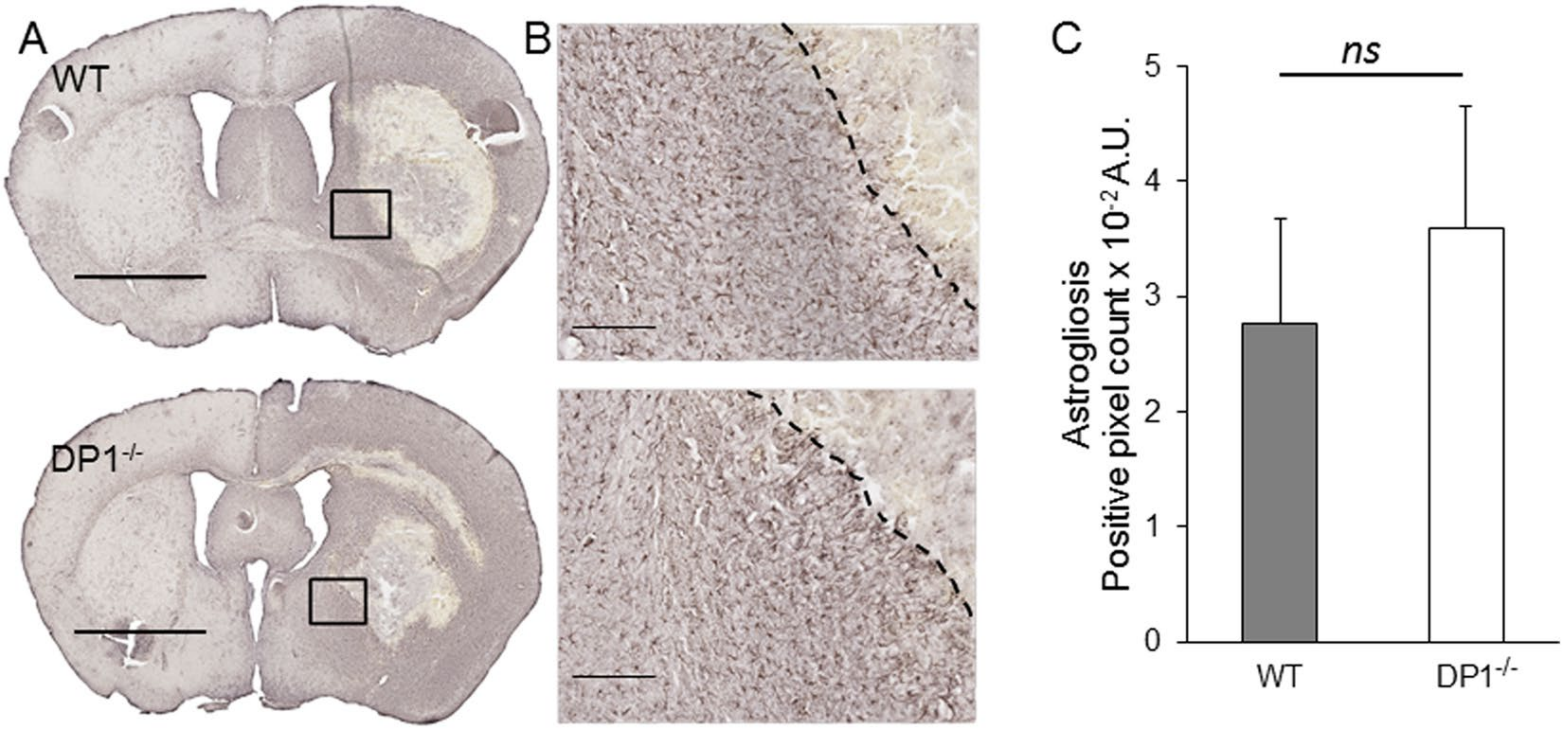
Astrogliosis remain unaffected by DP1 receptor deletion. The brain sections were immunostained for GFAP immunoreactivity to determine astrogliosis. (**A**) Representative macrographs at 1x showing GFAP immunoreactivity in WT and DP1^−/−^, mice. Scale bar = 2 mm. (**B**) The region in black boxes in panel A were magnified at 10x. The black dotted line demarcates the lesion core from the perihematoma area. No immunoreactivity toward astrocytes was detected in lesion core, whereas the perihematoma and intact area shows the astrocytes’ immunoreactivity. Scale bar = 200 µm. (**C**) Quantification of the GFAP immunoreactivity show no difference in WT and DP1^−/−^. ns = non-significant, *P* = 0.11. n = 8/group.

Harvested brain sections were immunostained for Iba-1 and GFAP immunoreactivity. The Iba-1 immunopositive cells were significantly higher in the ipsilateral side with predominant amoeboid or activated morphology in the preihematoma region. We found that DP1^−/−^ or WT treated with laropiprant has significantly lower Iba-1 immunoreactivity as compared with the WT mice (Fig. 3A and B). The immunoreactivity for Iba-1 decreased by 48.63 ± 24.40 and 47.94 ± 24.40 in DP1^−/−^ and laropiprant groups respectively (Fig. 3C). Two distinct morphological characteristics of Iba-1 immunopositive cells were detected representing classically activated microglial form and amoeboid shaped microglia/macrophages. Interestingly, there was strong GFAP immunoreactivity on the ipsilateral hemisphere along the perihematoma in both groups; however, there was no significant difference in immunoreactivity between the between the WT and DP1^−/−^ mice (Fig. 4A–C).

### Laropiprant Treatment or Deletion of DP1 Receptor Attenuates Iron Content

**Figure 5:**
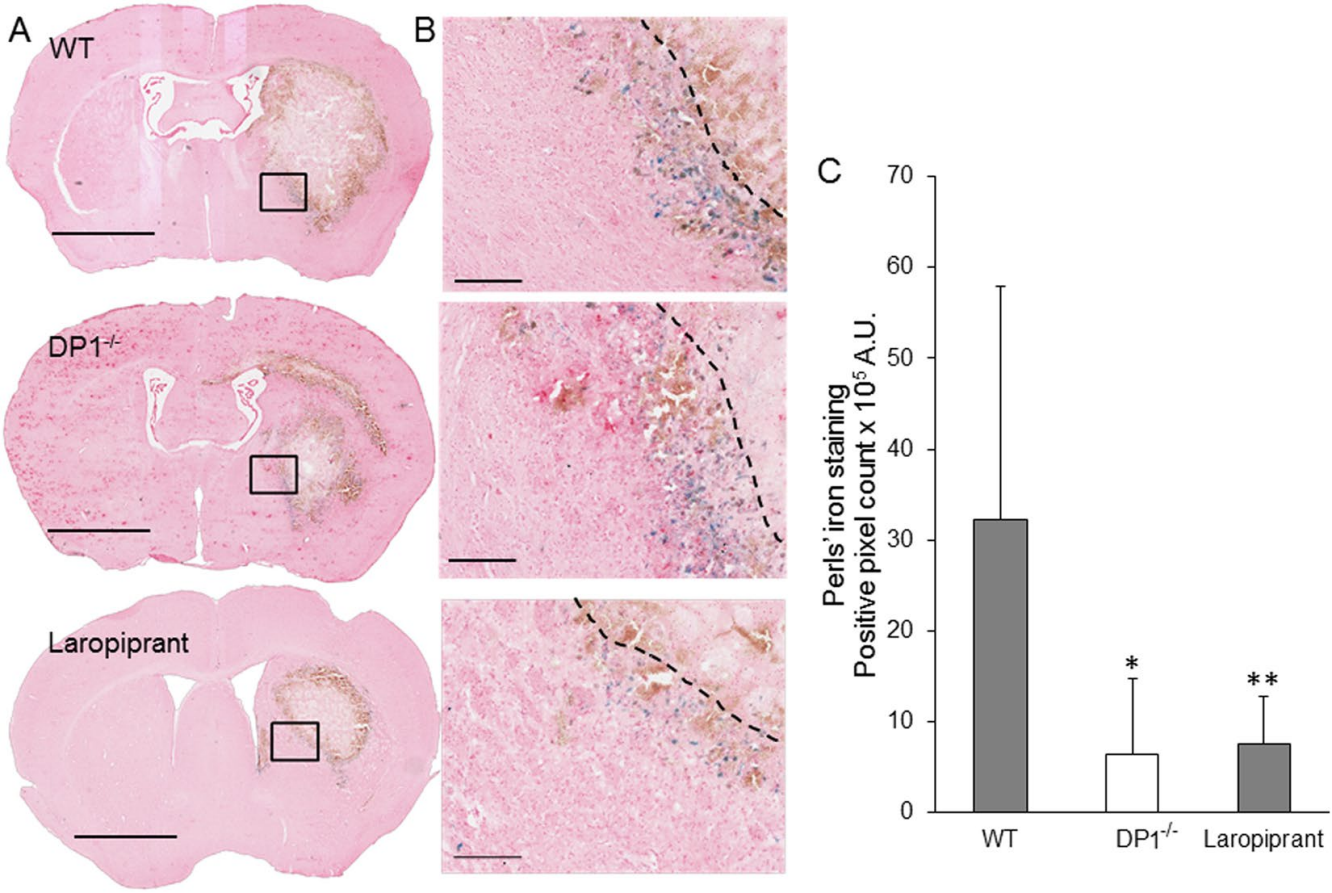
Laropiprant attenuates iron content in perihematoma. The brain sections were stained with Perls’ stain for iron content quantification. (**A**) Macrographs showing the sections obtained from WT, DP1^−/−^, and WT mice treated with laropiprant with Perls’ stain. Scale bar = 2 mm. (**B**) The regions in the black boxes in panel A were magnified at 10x. Blue dots represent iron staining, which was along the perihematoma region. Scale bar = 200 µm. Black dotted line demarcates lesion core from perihematoma area. (**C**) Quantification of Perls’ staining shows a significant decrease in iron content in DP1^−/−^ and laropiprant-treated groups. *P < 0.05, **P < 0.01 compared with the WT group. n = 6–7/group.

One of the major causes of secondary injury following ICH is hemolysis, which results in iron accumulation and augments neuroinflammation and oxidative stress. With Perls’ staining, the results suggest that DP1 receptor deletion or WT treatment with laropiprant resulted in a significant decrease in iron content (Fig. 5A and B). Such staining in DP1^−/−^ and laropiprant treatment groups decreased by 80.13 ± 26.03% and 76.25 ± 16.27%, respectively (Fig. 5C).

## Discussion

Intracerebral hemorrhage is a devastating acute neurological condition with high mortality and morbidity and limited treatment options. In this study, we used the selective PGD_2_ DP1 receptor antagonist laropiprant to test the hypothesis whether DP1 inhibition or absence will prevent hemorrhagic brain damage. We found that deletion of the DP1 receptor significantly attenuated the induced-ICH lesion volume, and similar effects were also observed with the laropiprant treatment. We also found that laropiprant augmented the *ex vivo* coagulation; whereas, deletion of the DP1 receptor or laropiprant treatment also attenuated the tail bleeding time, microgliosis, and iron content. These data support the usefulness of laropiprant as a therapeutic tool to resolve primary as well as secondary brain damage following ICH. We believe that this is the first study to show the beneficial effects of clinically tested laropiprant in ICH treatment.

Primary damage caused by ICH occurs within the first few hours and is mainly caused by bleeding and hematoma expansion, resulting in poor prognosis and high mortality. Immediate interventions to remove the hematoma or stop the bleeding are important therapeutic considerations. Therefore, one focus of this study was to test the feasibility of laropiprant in limiting bleeding during the initial phase of ICH. Interestingly, laropiprant has been tested in clinics for its role in niacin-induced flushing and nasal airway obstruction in humans^29, 40–42^; however, it has not been tested in neurologic conditions. Laropiprant is reported to abolish the PGD_2_-induced platelet inhibition^43^, thereby suggesting its potential in minimizing hemorrhage. Similarly, our data shows that treatment of ICH one hour after the onset can limit intracranial bleeding and lesion volume and improve neurologic deficits.

The first attempt to minimize ICH is to reduce the intracranial bleeding by regulating hemostasis, which is maintained through a network of processes including the platelet system, coagulation, and anticoagulant and fibrinolytic pathways^44–46^. The interaction of the downstream cascades of these pathways facilitates the adhesion of platelets with the sub-endothelial collagen in damaged vessels, forming a hemostatic vascular plug^47^. These plugs prevent further leakage of the blood into the brain parenchyma and limits hematoma expansion, consequently limiting secondary brain damage. Because PGD_2_ through DP1 receptor activation is reported to inhibit at least one of these processes^43^, we next tested whether DP1 receptor inhibition can limit bleeding and found that DP1 receptor inhibition or its deletion can indeed attenuate bleeding and limit ICH lesion volume. However, a few clinical trials indicate that attenuation of hematoma expansion or removal of a blood clot may not necessarily improve functional outcomes. For example, in clinical settings, the Surgical Trial in Intracerebral Hemorrhage (STICH) did not provide convincing evidence supporting the efficacy of early surgical removal of hematomas^48^. Similarly, use of recombinant activated factor VII did not improve survival or functional outcomes in ICH patients, although it did successfully reduce hematoma enlargement^49^. Nevertheless, the observations in our current study indicate that laropiprant treatment may have broader therapeutic effects. Such observation requires further investigation by testing the effect of laropiprant on long-term outcomes. Moreover, the observed Perls’ staining data also suggests that there is lower iron content in DP1^−/−^ mice and WT mice treated with laropiprant. It is yet to be confirmed whether the lower iron levels in DP1^−/−^ or laropiprant treatment groups are due to the restricted bleeding or if they are a result of the better microglial phagocytosis in DP1^−/−^ and laropiprant treatment groups than in WT mice, as the role of microglial phagocytosis in hematoma clearance has been reported.

The current study supports a clear role of laropiprant in attenuating ICH-induced brain injury by limiting hematoma expansion; however, its efficacy for human use has been potentially challenged by a recent clinical trial. The HPS2-THRIVE trial was conducted to investigate the favorable effects of niacin/laropiprant combination to supplement the beneficial effects of statin in minimizing cardiovascular events. However, the trial concluded that this combination did not have a useful beneficial effect, but rather produced some side effects including hemorrhage in some cases^28, 50^. Careful review of the published data suggests that the side effects observed in the HPS2-THRIVE trial could be due to the fact that both niacin and statin are vasodilators and could have additive or synergistic effects leading to exacerbated hemorrhagic conditions^40, 51–53^. Moreover, in an attempt to achieve the pharmacologic dose that can minimize the niacin-induced flushing, the trial may have used higher doses (20 or 40 mg) of laropiprant rather than testing its lower effective dose^26^. It was reported that a dose of 6 mg or higher effectively suppressed PGD_2_-induced platelets aggregation. It is noteworthy that at higher concentrations, laropiprant could potentially also act as a TxA_2_ TP receptor antagonist^54^, though no clinically significant effect on bleeding times was observed with multiple doses of laropiprant alone adding up to 200 mg^26^. However, it is likely that the combination of high dose of laropiprant with niacin may have had an additive effect with statin in the HPS2-THRIVE trial, leading to hemorrhagic conditions. Interestingly, in our models, we found that 0.4 mg/kg, which is equivalent to 1.94 mg for a 60-kg human based on the body surface area dose conversion method^55^, had a protective effect against ICH. Furthermore, we also tested whether sub-chronic treatment of laropiprant could lead to microthrombosis or microhemorrhage in non-ICH naïve WT mice. Estimation of brain hemoglobin content on day 7 of the laropiprant treatment reveals no difference in the optical density of the brain samples obtained from vehicle or laropiprant-treated groups suggesting that laropiprant has no detrimental effect on hemostasis under non-pathologic (i.e. non-hemorrhagic) conditions.

The beneficial effects of laropiprant observed here could also be through regulating secondary injuries such as neuroinflammation. Microglial activation in response to injury could have beneficial or detrimental effects. Data from various labs, including ours, suggest that microglial activation in ICH results in phagocytosis and hematoma clearance^11, 19, 56^. However, generally chronic microglial activation is associated with increased inflammation and augmented brain damage^25, 57, 58^. Under neurologic conditions activated microglia produce proinflammatory cytokines such as TNFα and IL-1β, reactive oxygen/nitrogen species, and chemokines thereby augments brain damage^16, 59, 60^. Interestingly, our current data shows that DP1 receptor inhibition decreases microglial activation (Fig. 3). This decrease could be a result of lower lesion volume or lower iron content which then resulted in lower neuroinflammation. Interestingly, there was strong GFAP immunoreactivity in the ipsilateral side; however there was no difference between the WT and DP1^−/−^ mice groups.

### Limitations

This study shows for the first time proof-of-concept that DP1 antagonism, by clinically administered drug, could limit hematoma expansion and ICH brain damage. However, there are certain limitations that warrant further investigation. For example, (1) the clinical dose with and without niacin combination also needs to be investigated in ICH condition. (2) Because often older populations or populations with comorbidities are at greater risk of ICH, similar studies in older animals and animals with comorbidities such as diabetes or hypertension will provide more information regarding the therapeutic benefits of laropiprant. (3) To test the proof of the concept and to avoid any confounding effects due to sex differences, only male mice were used in this study. Therefore, additional studies are needed to test the therapeutic efficacy of laropiprant in young and old females. (4) Although we found that laropiprant affects bleeding and regulates hematoma volume, a detailed study on hemostasis would provide mechanisms associated with laropiprant treatment. (5) We also reported attenuated microgliosis in laropiprant treatment groups; however, a detailed study on acute and chronic effects of laropiprant on macrophage and microglial polarization will provide additional associated mechanisms.

## Conclusions

Our data reveal for the first time that DP1 receptor inhibition or deletion is beneficial against ICH. Although in the recent trials, use of laropiprant with niacin along with statin therapy did not provide the desired beneficial effects, the efficacy of laropiprant alone in minimizing intracranial bleeding can still be considered a vital therapeutic strategy. To draw more reliable conclusions, further studies are needed to investigate the mechanism(s) associated with laropiprant treatment and its role in regulating primary and secondary brain injury following ICH.
